# Biallelic Mismatch Repair Deficiency in an Adolescent Female

**DOI:** 10.1155/2018/8657823

**Published:** 2018-07-25

**Authors:** Amber Hildreth, Mark A. Valasek, Irene Thung, Thomas Savides, Mamata Sivagnanam, Sonia Ramamoorthy, Sherry C. Huang

**Affiliations:** ^1^University of California San Diego Department of Pediatrics, Division of Gastroenterology, USA; ^2^Rady Children's Hospital San Diego, USA; ^3^University of California San Diego Department of Pathology, USA; ^4^University of California San Diego Department of Medicine, Division of Gastroenterology, USA; ^5^University of California San Diego Department of Surgery, Division of Colorectal Surgery, USA

## Abstract

Constitutional (Biallelic) Mismatch Repair Deficiency is a rare autosomal recessive disorder characterized by numerous cancers presenting as early as the first decade of life. Biallelic germline variants in one of four mismatch repair genes (*MLH1, MSH2, MSH6, *or* PMS2*) cause this devastating disease. Given the rarity of the syndrome, often-asymptomatic tumors, and overlap with neurofibromatosis-1, diagnosis is frequently unrecognized or delayed. We present a unique case of a 14-year-old female with minimal gastrointestinal symptoms diagnosed with invasive adenocarcinoma secondary to biallelic* PMS2 *variants.

## 1. Introduction

Constitutional (Biallelic) Mismatch Repair Deficiency (BMMRD) is a rare autosomal recessive disorder characterized by early-onset cancers as early as the first decade of life. It is caused by inherited biallelic mismatch repair (MMR) gene variants, most commonly* PMS2* [1–4]. This is in contrast to Lynch Syndrome (LS), the most common cause of hereditary adult-onset colorectal cancer, where patients have a heterozygous variant in one of the MMR genes [5, 6]. Affected individuals have been described with childhood onset of gastrointestinal tumors, brain tumors, and hematologic malignancies, as well as rare cases of urologic malignancies [7]. While up to two-thirds of patients present with colonic tumors, diagnosis is difficult, as they do not often present with painless rectal bleeding as seen with other pediatric polyposis syndromes [8, 9]. Notable defining features of this disease include café-au-lait macules similar to neurofibromatosis-1, found in the majority of patients, and a family history of consanguinity, which has been described in approximately half the reported diagnoses [5]. We present a unique case of a 14-year-old female diagnosed with invasive adenocarcinoma, lacking classical BMMRD features, found to have biallelic pathogenic* PMS2* mutations.

## 2. Case Presentation

The proband is a 14-year-old previously healthy female born to nonconsanguineous healthy parents who was admitted for having fever, fatigue, lower quadrant abdominal pain, and vomiting. Abdominal computerized tomography (CT) revealed significant hydronephrosis consistent with a right ureteropelvic junction (UPJ) obstruction, for which she underwent ureteral stent placement on hospital day 2. Her postoperative course was complicated by continued abdominal pain and fever, as well as an episode of rectal prolapse. Of note, she also endorsed an episode of rectal prolapse months prior to presentation which was manually reduced at home. On hospital day 6, due to continued fevers, abdominal and pelvic MRI was obtained. This study revealed a pelvic fluid collection concerning abscess; CT guided transgluteal drainage performed by Interventional Radiology resulted in 150ml cloudy yellow fluid. The etiology was believed to be an infected urinoma caused by instrumentation during stent placement and she was treated with ceftriaxone and metronidazole. The patient's fevers and pain continued, prompting an abdominal and pelvic CT on hospital day 10. The imaging was notable for diffuse ascites, bowel wall thickening, and organizing fluid collections within the pelvis. She ultimately went for exploratory laparotomy and washout with intraoperative findings of multiple pockets of turbid fluid. The entire bowel was evaluated and there was no evidence of perforations, fistulas, or other causes of gastrointestinal leakage. Peritoneal fluid cultures grew polymicrobial organisms, however, suggestive of gut flora. She was transitioned to meropenem and fevers eventually resolved prior to discharge.

**Figure 1:**
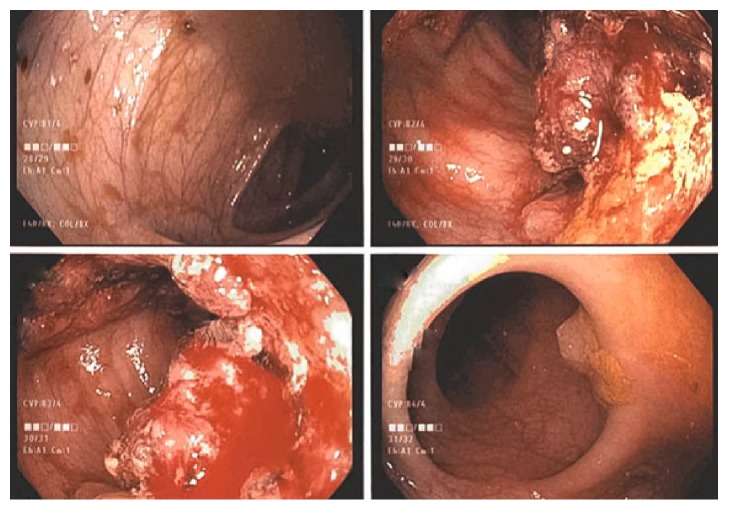
Endoscopic photographs showing normal colonic mucosa (top left), abnormal polypoid mass (top right and bottom left), and sessile polyp (bottom right).

**Figure 2:**
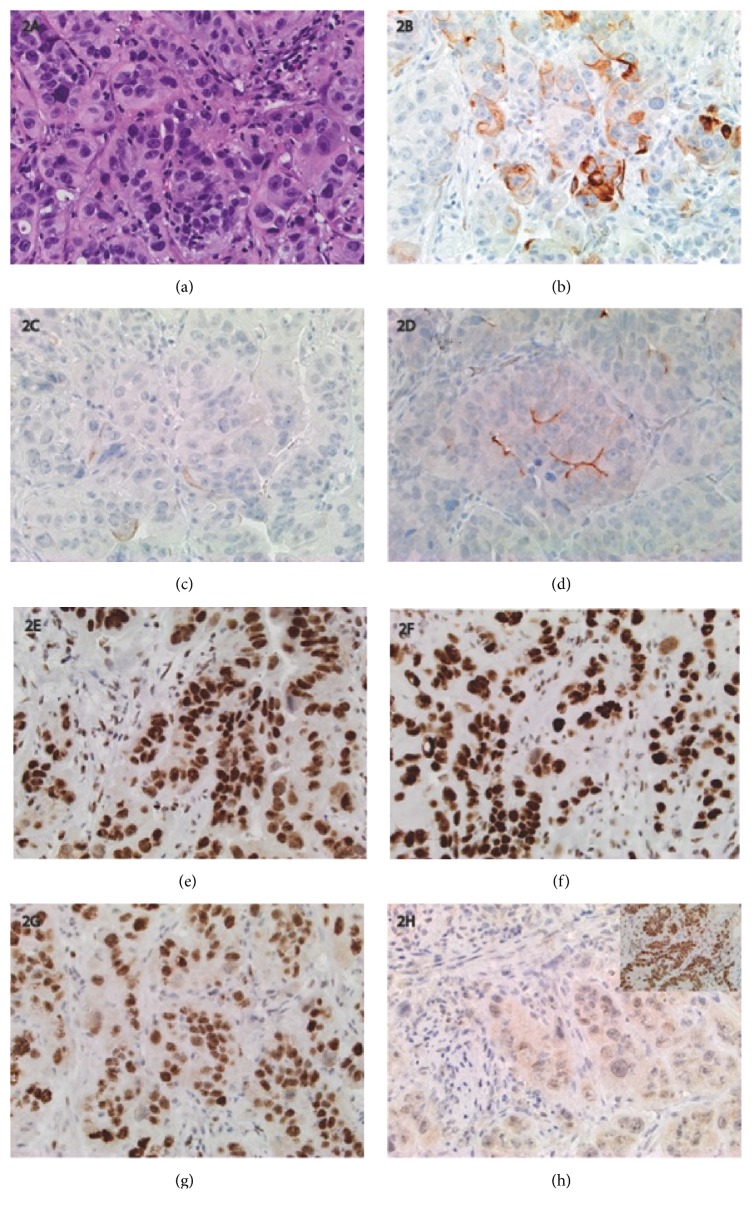
Immunohistochemical studies for PMS2 deficient adenocarcinoma, 40X. (a) H&E: moderately differentiated adenocarcinoma with crowded glands and minimal stroma. (b) CK7: focal positive immunostaining for CK7. (c) CK20: negative immunostaining for CK20. (d) Villin: focal minimal superficial (brush border) immunostaining for villin. CDX2 was also focally positive (not shown). (e) MLH1: intact nuclear immunostaining for MLH1 (no loss of expression). (f) MSH2: intact nuclear immunostaining for MSH2 (no loss of expression). (g) MSH6: intact nuclear immunostaining for MSH6 (no loss of expression). (h) PMS2: loss of nuclear immunostaining for PMS2 in both tumoral cells and background lymphocytes. Positive tissue control stains were appropriately positive (inset).

Two months following discharge the patient underwent outpatient elective appendectomy, right pyeloplasty, and ureterotomy with stent placement due to persistent hydronephrosis. Pathology revealed normal appendix tissue. Ureter pathology was significant only for acute and chronic inflammation, with no evidence of malignancy. Routine screening abdominal ultrasound 2 months later revealed stable hydronephrosis; however, an incidental 3cm soft tissue mass presumed to be near the sigmoid colon was noted. Follow-up MRI showed nearly circumferential thickening of the sigmoid colon which was subsequently evaluated by esophagogastroduodenoscopy and colonoscopy. Findings were significant for 9-10 sessile polyps in transverse, descending, and sigmoid colon with large cluster of irregular appearing polyps in the sigmoid (Figure 1). Histologic evaluation of the sigmoid polyp revealed invasive moderately differentiated adenocarcinoma with extensive lymphovascular invasion. Immunohistochemical staining demonstrated the carcinoma to be positive for CDX-2, villin, and CK7 and negative for CK20 and WT-1 (Figure 2). The lack of CK20 suggested a possible primary upper gastrointestinal or hepatobiliary primary tumor; however this was not found on endoscopy or suggested on previous imaging. Further immunohistochemical staining revealed no loss of expression of* MLH1, MSH2, *and* MSH6. *There was complete loss of expression of* PMS2*; this testing was deemed indeterminate due to lack of staining in both tumor tissues and normal tissue.

The patient underwent repeat colonoscopy approximately 3 weeks after her first procedure with an adult interventional gastroenterologist. Findings were significant for two tubular adenomas in the colon at 25cm and 30cm from the anus, moderately differentiated adenocarcinoma at 24cm, a moderately differentiated adenocarcinoma in the rectum (10-15cm), and an anorectal mass with moderately differentiated adenocarcinoma arising in the background of tubulovillous adenoma with extensive high-grade dysplasia. Both tubular adenomas were removed by snare polypectomy, while the remaining masses were biopsied. Immunohistochemical staining was positive for CDX2, CK7, CK20, and villin. There was no loss of expression of* MLH1*,* MSH2*, and* MSH6*, suggesting intact proteins. There was again loss of expression of* PMS2* in both tumor and nontumoral cells (Figure 2) consistent with complete protein loss. Genetic testing revealed biallelic pathogenic* PMS2* variants: NM 000535: c.137 G>T; NP 0005261: pS46I and c.1831dupA (frameshift). Familial testing concluded that one variant was inherited from each parent, with her asymptomatic brother also carrying the father's* PMS2* variant.

Due to the findings of invasive disease as well as previous pelvic fluid suggestive of a microperforation, the diagnosis of Stage 4 adenocarcinoma was established. Chemotherapy was initiated with Capecitabine (pyrimidine analog) followed by two cycles of FOLFOX6 (Oxaliplatin, Leucovorin, and 5-FU), as well as 6 weeks of radiation. She finally underwent total colectomy along with total abdominal hysterectomy with bilateral salpingectomy, and pathology revealed no evidence of residual adenocarcinoma in the colon.

## 3. Discussion

Although colorectal cancer in children is rare, this case demonstrates that invasive disease can be diagnosed in the pediatric population. While GI manifestations of BMMRD are well described and fit the phenotype of our patient's numerous adenomas and adenocarcinomas, our patient's only true gastrointestinal symptom was rectal prolapse, making her diagnosis even more challenging until incidental findings on ultrasound. More importantly, she lacked the most common features of familial consanguinity or café-au-lait macules which would trigger further investigation into genetic disease. It is extremely important to recognize BMMRD given its association with additional cancers including brain tumors, hematologic malignancies, and rare cases of urologic malignancies (two previous cases of bladder/ureter malignancies and two cases of ureter/renal pelvis malignancies have been described). The European consortium “Care 4 CMMRD” published guidelines for diagnosis in 2014 [10]. Data was based on the knowledge of the disease from just 146 known patients. In 2017 Durno and colleagues expanded upon these guidelines with further diagnostic and surveillance recommendations for this vulnerable population [11]. Once the diagnosis of BMMRD was established in our patient, renal involvement was also considered given her initial presentation of a UPJ obstruction; however, review of ureteral pathology showed no evidence of malignancy.

Biallelic mutations have been reported in all four DNA MMR genes (*MLH1, MSH2*,* MSH6, *and* PMS2*). However, the majority of BMMRD patients with GI cancers report biallelic mutations in* PMS2*. In classic LS, monoallelic carriers of* PMS2 *variants have a lower penetrance for GI cancers. The combination of the early-onset cancer in the proband, later-onset cancer in the parents, and incomplete penetrance for* PMS2*-LS leads to a family history that is often negative [11]. This may also explain how two unrelated healthy individuals with heterozygote* PMS2* variants would remain unaffected and produced offspring without being aware of his/her own risk of cancer development. Our case is unique in the fact that there was no consanguinity in the family. Forty-six of the 91 known families with BMMRD demonstrate either consanguinity or homozygosity for one mutation [10]. It is pivotal to identify the genetic cause in patients and their family members as it provides information pertinent to lifelong medical screening. In the end, it is paramount for clinicians to always recognize the potential for a rare disease diagnosis both to provide the best care for our patients and to expand the knowledge and recognition of disorders such as BMMRD.
